# Characteristics of patients who received helicopter emergency medical services in Japan from 2012 to 2019: a retrospective analysis of data from Tochigi Prefecture

**DOI:** 10.1186/s13049-022-01012-6

**Published:** 2022-04-11

**Authors:** Koji Wake, Takafumi Noguchi, Hidekazu Hishinuma, Masayoshi Zaitsu, Jin Kikuchi, Masatoshi Uchida, Kentaro Hayashi, Masanari Machida, Hajime Houzumi, Eisei Hoshiyama, Kyo Takahashi, Gen Kobashi, Kazuyuki Ono

**Affiliations:** 1 Department of Emergency and Critical Care Medicine, Dokkyo Medical University, Shimotsuga-gun, Tochigi Japan; 2 Department of Public Health, School of Medicine, Dokkyo Medical University, Shimotsuga-gun, Tochigi Japan; 3 Department of Adult Nursing, Dokkyo Medical University School of Nursing, Shimotsuga-gun, Tochigi Japan; 4 Department of Neurology, Dokkyo Medical University, Shimotsuga-gun, Tochigi Japan

**Keywords:** Helicopter emergency medical services, Pre-hospital intervention, Doctor-Heli, Ambulance

## Abstract

**Background:**

Helicopter Emergency Medical Services (HEMS) has been in operation in Japan since 2001, allowing patients almost anywhere in the nation to receive on-scene emergency treatment from physicians. However, there is insufficient literature on the characteristics of the patients who use Japanese HEMS. Thus, this study aimed to investigate the overall characteristics of patients receiving HEMS care within a single prefecture in Japan.

**Methods:**

We retrospectively analyzed the data of 5163 patients—in Tochigi Prefecture—who received HEMS care from 2012 to 2019. Descriptive statistics were used to analyze the following aspects of care: diagnosis, severity, background characteristics, geographical and environmental variables, immediate pre-hospital intervention, transportation type, and short-term clinical outcomes.

**Results:**

Among 7370 HEMS requests received during the study period, treatment was provided to 5163 patients (1.8 cases per day; 3489 men [67.6%]). Nearly 55% (n = 2856) of patients were aged above 60 years. Age peaks were observed at 0–9 years and 60–69 years. The median distance from the base hospital to the site was 26.7 km. The age-standardized rate of HEMS treatment was 30.3 patients per 100,000 people. Cases of trauma and cardiovascular diseases were the most common (65.3%). Most individuals aged 0–9 years and 60–69 years had neurological disease (seizures accounted for 80.5% of this group) and cardiovascular disease, respectively. The number of patients was similar across all four seasons. After immediate pre-hospital intervention, 81.6% of patients receiving HEMS care were transferred by the helicopter ambulance (53.4% and 28.2% to the base hospital and to other hospitals, respectively). Overall, 56.6% of patients receiving HEMS care were transferred to the base hospital, and the short-term recovery rate was above 75%. Intravenous drip and oxygen administration were the most common pre-hospital interventions (93.1% and 72.7%, respectively).

**Conclusions:**

This study is the first to describe the overall characteristics of HEMS patients using comprehensive data of all HEMS patients in one prefecture in Japan. Further research using both local- and national-level data is needed to accelerate the understanding of the benefits of HEMS.

**Supplementary Information:**

The online version contains supplementary material available at 10.1186/s13049-022-01012-6.

## Background

Helicopter emergency medical services (HEMS), which was initially launched in Germany and the U.S.A. in 1970, has now been adopted worldwide to meet the needs of urgent and severe emergency conditions [1–3]. Globally, approximately 2750 helicopters associated with HEMS provide emergency medical care for exogenous emergencies (e.g., trauma, toxicosis, and anaphylaxis) and endogenous emergencies (e.g., cardiovascular diseases) within 30 min.

In Japan, HEMS was established in 2001, with a helicopter ambulance called “Doctor-Heli.” Generally, the ambulance staff of ground emergency medical services are prohibited from providing advanced medical care, such as ultrasound examination, endotracheal intubation, and pleural drainage (except for onboard physicians in a special automotive ambulance, called “Doctor-Car”). However, HEMS emergency physicians travel onboard to provide immediate on-scene treatment, including advanced medical care [3]. While the Japanese Ministry of Health, Labour and Welfare (MHLW) is responsible for managing HEMS, a public service, the operations are carried out by local governments. Designated helicopter base hospitals manage emergency physicians and nurses onboard, whereas the commercial companies commissioned by these base hospitals manage mechanical staff (e.g., helicopter captains, mechanics, and communicators) and maintain helicopter ambulances. As of April 2021, 54 helicopter ambulances (95.7%) were on standby in 45 out of 47 prefectures in Japan, which means that emergency patients almost everywhere in Japan can be theoretically reached by emergency physicians within 30 min [3].

Although HEMS is recognized as an essential system in emergency medicine, a recent systematic review did not conclude that the HEMS benefits survival and/or mortality [4]. In Japan, evidence has been limited to specific emergency conditions (e.g., cardiovascular diseases and trauma) [5–12]. However, the overall characteristics of the Japanese HEMS patients who receive immediate on-scene treatment remain unclear. Therefore, to expedite further understanding of the current HEMS situation in Japan, a descriptive epidemiology—of all emergency diseases and trauma—with a large-scale database is essential [13, 14].

The purpose of this study was to describe the overall characteristics of HEMS patients using comprehensive data of all HEMS patients in one prefecture in Japan.

## Methods

### Aim

The aim of the current study to describe the overall characteristics of HEMS patients using comprehensive data of all HEMS patients in Tochigi prefecture in Japan.

### Study design, setting, and participant characteristics

In this retrospective and descriptive study, we analyzed the data of all patients who utilized HEMS in Tochigi across a span of eight years (2012–2019). The details of Tochigi Prefecture have been outlined in previous studies [11, 15]. Briefly, Tochigi Prefecture has 1.9 million people (1.5% of Japan's total population) with an area of 6408 km^2^, stretching across approximately 84 km east–west and 98 km north–south. It is located 100 km to the north of Tokyo. The age and sex distributions in the region are similar to the national age and sex distributions, respectively. The major industries in the prefecture are manufacturing, agriculture, and forestry, and the area is surrounded by mountains with no area facing the sea. The Tochigi HEMS started operating in January 2010 with its base hospital being Dokkyo Medical University Hospital.

**Fig. 1:**
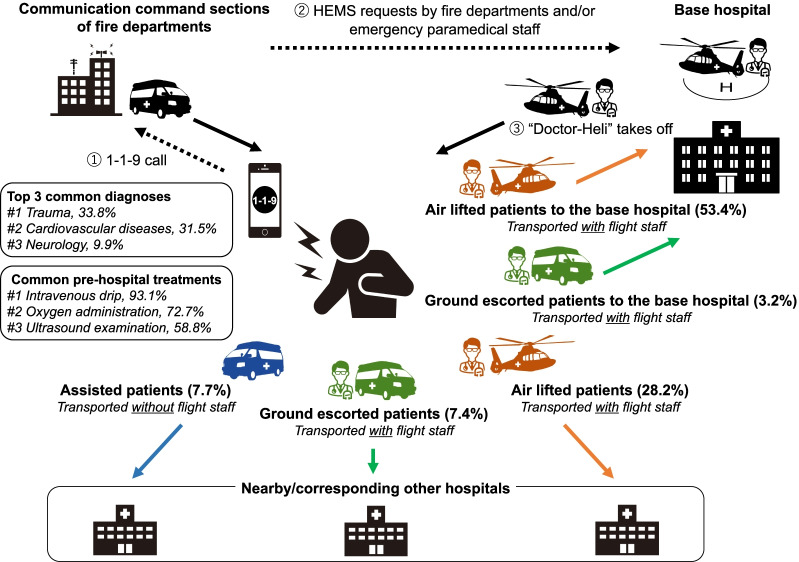
Japanese HEMS request and transportation system and corresponding statistics of Tochigi prefecture. A helicopter ambulance is called “Doctor-Heli” in Japan. The air lifted or ground escorted patients are either transported to the base hospital or to other hospitals, accompanied by the onboard emergency physician. In contrast, the assisted patients who do not require the attendance of the onboard emergency physician after the initial treatment are transported to the nearest available hospital by the automotive ambulance. Percentages may not total 100 because of rounding. Note: HEMS, helicopter emergency medical services

The Tochigi HEMS follows the general rule for HEMS in Japan, which states that patients cannot request the HEMS directly (Fig. 1). Communication needs to be routed via the fire department and/or emergency paramedical staff, who can explicitly request the HEMS in the following cases: (1) immediately upon receipt of a 1-1-9 call, (2) on the way to the scene, or (3) after the initial on-scene assessment and systemic observation. Once HEMS is called for, the following protocol is generally adopted: (1) the helicopter ambulance takes off within 3 min, (2) an automotive ambulance without emergency physicians simultaneously transports the patient from the incident site to the helicopter landing point, (3) the helicopter ambulance lands at the landing point that is nearest to the patient from among approximately 600 designated landing points, (4) onboard emergency physicians and nurses begin primary treatment immediately upon arrival, (5) onboard emergency physicians either choose the helicopter or automotive ambulance to transport the patient to the best suitable designated hospital based on the diagnosis or treatment requirements [11].

The helicopter ambulance, which can reach anywhere in the prefecture within 20 min, is kept on standby at the base hospital, and the dispatch criteria are shown in Table 1. In addition, in July 2011, Tochigi Prefecture made an agreement of Northern Metropolitan Wide-Area Cooperation with two neighboring prefectures—Ibaraki Prefecture on its east Gunma Prefecture in the west—in the northern metropolitan area. Thus, if the Tochigi helicopter ambulance is unavailable, the Tochigi emergency medical services can either request the Ibaraki or Gunma helicopter ambulances that are on standby. Similarly, if helicopters in Ibaraki or Gunma prefecture are unavailable, they can call for a helicopter from Tochigi. However, the range of dispatches is restricted to a radius of approximately 50 km from each base hospital; therefore, the Ibaraki cannot call for a helicopter from Gunma prefecture and vice-versa.

**Table 1:** Dispatch criteria for the HEMS in Japan

1. Due to trauma	
(1) Severe trauma	
① High-energy trauma (such as vehicle accidents with passenger fatalities, off-board release, crashing from a height)	
② Multiple trauma	
③ Obvious abnormality in vital signs (such as consciousness, breathing, blood pressure, pulse rate, and body temperature)	
Trauma	
④ Penetrating trauma (such as puncture wounds, gunshot wounds, etc.)	
⑤ Trauma with marked external bleeding	
⑥ Finger amputation	
(2) Severe burn	
① Burns over 15% of the body surface area	
② Airway burns (including consciousness disorder, facial burns, injuries in enclosed spaces, etc.)	
③ Chemical burns	
④ Burns accompanied by external injuries (due to explosions)	
(3) Drowning and suffocation	
(4) Acute toxicosis	
① Acute drug toxicosis (such as antidepressants, lithium carbonate, beta-blockers, calcium antagonists, acetaminophen)	
② Carbon monoxide toxicosis	
(5) Anaphylaxis	
(6) Environmental obstacle(s)	
Decompression sickness, accidental hypothermia, heatstroke, etc.	
2. Due to disease(s)	
(1) Consciousness disorder, convulsion, paralysis, and/or strong headache (including stroke, etc.)	
(2) Strong chest pain and/or abdominal pain (including myocardial infarction, aortic disease, etc.)	
(3) Dyspnea (including bronchial asthma, acute heart failure, etc.)	
(4) Obvious abnormality in vital signs (such as consciousness, respiration, blood pressure, pulse rate, and body temperature)	
3. Cardiopulmonary arrest	
(1) Case of cardiopulmonary arrest in which the heartbeat is restarted by cardiopulmonary resuscitation (CPR)	
(2) Case of cardiopulmonary arrest in which the first electrocardiogram shows ventricular fibrillation or tachycardia (VT/VF) or pulseless electrical activity (PEA)	
(3) Case of cardiopulmonary arrest in which the instructing doctor judges that he is suitable for Doctor-Heli during online medical control	
4. Perinatal emergency disease(s)	
5. Others judged to be serious in the field-instructed	
6. Case in which the instructing doctor judges that he is suitable for Doctor-Heli during online medical control	

The Tochigi HEMS database includes basic information such as sex, age, and date of HEMS request. It also includes clinical information such as pre-hospital diagnosis, severity, and treatment. The geographical information included on-site points and duration of initial contact with the patient from the time of the call for service. The database uniquely includes short-term clinical outcomes at the end of the initial treatment in the emergency room of the base hospital. Data for ground emergency medical services were not included in the database. The accuracy and quality of the dataset has been maintained by medical staff involved in Tochigi HEMS. We obtained de-identified data under the research agreement between the authors and the Tochigi HEMS. The study was carried out in accordance with the guidelines outlined in the Helsinki Declaration of 1964; the study was approved by the research ethics committee of Dokkyo Medical University Hospital (Protocol Number R37-21J).

**Fig. 2:**
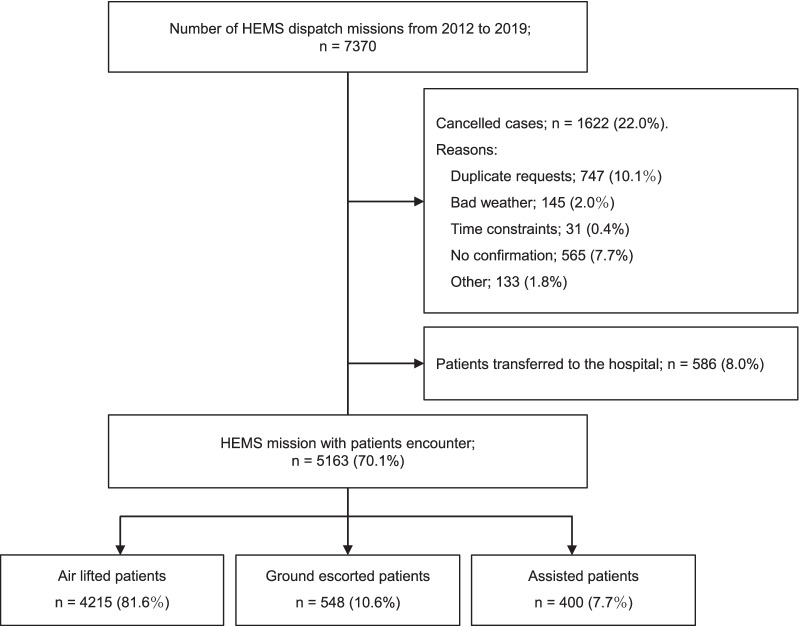
Flowchart showing all the Tochigi Helicopter Emergency Medical Service missions and inclusion of patients. Of 7370 HEMS dispatch missions (2012–2019), 5163 cases were included in the analysis

Of the 7370 patients for whom the HEMS was requested during the study period, we excluded 22.0% of patients owing to non-matching conditions for dispatch (n = 1621) and 8.0% patients who were inter-hospital transfers (n = 586). Finally, data of 5163 patients were included in the analyses (Fig. 2).

### Definitions of diagnosis and severity

The patients were classified into nine groups: trauma; neurology; cardiovascular diseases (including ischemic heart disease, aortic disease, and stroke); cardiopulmonary arrest; respiratory disease; gastroenterology; allergies; toxicosis; and other diseases (such as burn, heatstroke, and threatened preterm labor). The measures that we used to classify the pre-hospital severity of each case included a simple severity classification (mild, moderate, severe, and death) by on-scene emergency physicians; it is commonly used in the practice of emergency medicine in Japan. The pre-hospital Glasgow Coma Scale (GCS) score (mild, 14–15; moderate, 9–13; severe, 3–8) determined by on-scene emergency physicians was also considered; however, other relevant indicators of the severity of the HEMS patients, such as the National Advisory Committee on Aeronautics (NACA) score, were unavailable.

### Background characteristics and geographical and environmental variables

Basic characteristics of the patients included sex, age (at the time of diagnosis), and year of diagnosis.

**Fig. 3:**
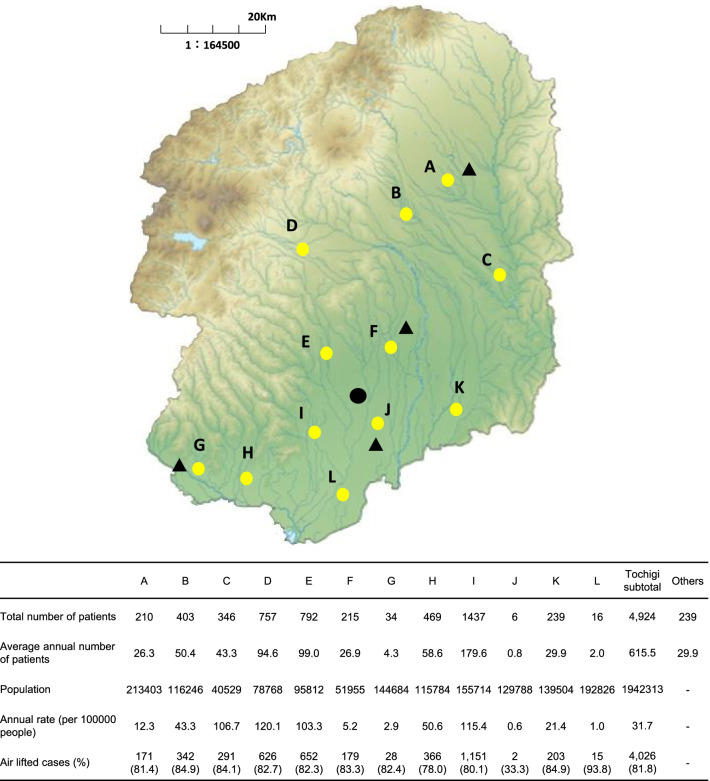
Numbers of patients in regional fire departments and their corresponding regional communication command section. Black circles indicate the base hospital (Dokkyo Medical University Hospital), triangles indicate the other four tertiary emergency centers, and yellow circles indicate the 12-communication command sections of fire department. The base hospital is mainly responsible for medical controlling in the D, E, and I areas, while the J area is jointly controlled by the base hospital and a tertiary emergency center. “Others” were those who requested Tochigi HEMS from two neighboring prefectures on the east (Ibaraki) and west (Gunma) by virtue of the agreement of Northern Metropolitan Wide-Area Cooperation

The geographical information included the calculated distance (in kilometers) from the base hospital to the designated landing point(s) in the prefecture [11]. We also grouped patients into 12 regional fire department areas with a regional communication command section (A to L; Fig. 3). Various arrangements are made in the medical control system in Japan for the overall coordination of emergency medical care in respective areas of emergency medical services [16]. Thus, the emergency center of the base hospital is mainly responsible for the medical control of D, E, and I (Fig. 3). The environmental variables included seasons: spring, summer, fall, and winter.

### Immediate pre-hospital intervention

Immediate pre-hospital intervention provided to the HEMS patients included intravenous drip, oxygen administration, ultrasound examination, endotracheal intubation, pleural drainage, chest compression, and external defibrillation.

### Mode and type of transport

In Japanese HEMS, the onboard emergency physician chooses either the helicopter ambulance (air lifted patients) or the automotive ambulance (ground escorted patients or assisted patients) to transport the patient to a hospital after the immediate pre-hospital intervention. The air lifted or ground escorted patients are either transported to the base hospital or to other hospitals, accompanied by the onboard emergency physician (Fig. 1). In contrast, assisted patients who do not require the attendance of the onboard emergency physician after the initial treatment are transported to the nearest available hospital by the automotive ambulance (Fig. 1).

### Short-term clinical outcome

For a supplementary data analysis, we assessed short-term clinical outcomes for the air lifted group transported to the base hospital, which were defined as changes in severity status from the time of initial contact (at the scene) to the end of treatment (in the emergency room) [11]. This short-term outcome, as confirmed by emergency physicians, was classified into four categories (recovery, no change, worse, and death). However, it was only available for those transported to the base hospital.

### Statistical analysis

Descriptive statistics were used for data analysis. Age (at the time of diagnosis) and time were reported as means and standard deviations; data for geographical distance were represented using medians and interquartile ranges. Other variables were represented as percentages. Although our data were based on the entire survey of one prefecture and were relatively sizable, we estimated the age-standardized rate of HEMS patients per 100,000 people in Tochigi Prefecture to facilitate the generalizability and comparability. We considered the 1985 model Japanese population and the mean annual HEMS cases in each age category during the study period.

## Results

During the study period of eight years, the total number of HEMS requests was 7370 (approximately 2.5 requests per day), whereas the number of HEMS patients treated on-scene by the HEMS system was 5163, which is an average of 1.8 cases per day (Fig. 2).

### Patient characteristics and distribution of diseases

**Fig. 4:**
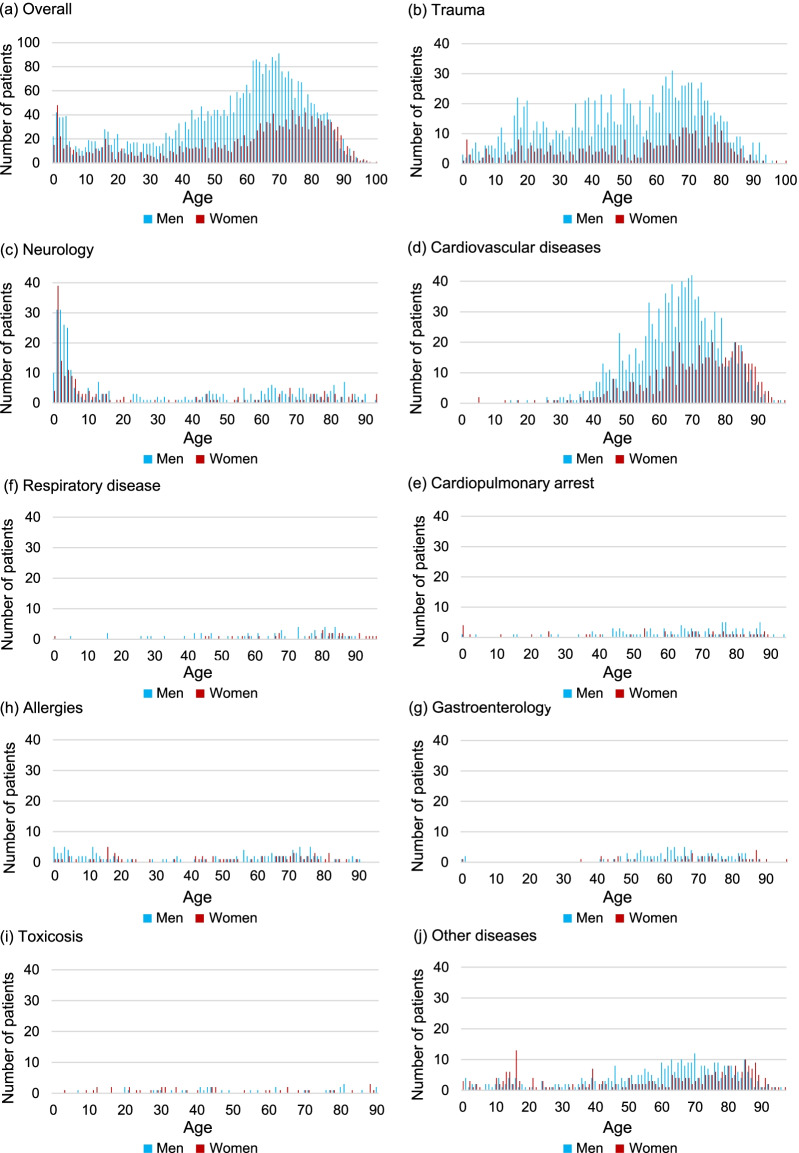
Disease-wise age distribution of patients in the Helicopter Emergency Medical Services. The numbers of patients were as follows: **a** overall, 5163 (men, 3489, women, 1674); **b** trauma, 1746 (men, 1300, women, 446; **c** neurology, 512 (men, 325, women, 187); **d** cardiovascular diseases, 1626 (men, 1081, women, 545); **e** cardiopulmonary arrest, 143 (men, 99, women, 44); **f** respiratory disease, 89 (men, 58, women, 31); **g** gastroenterology, 141 (men, 98, women, 43); **h** allergies, 196 (men, 125, women, 71); **i** toxicosis, 77 (men, 36, women, 41); **j** other diseases, 633 (men, 367 women, 266)

Baseline patient characteristics are shown in Table 2. Of the 5163 HEMS patients, 3489 (67.6%) were men and 2856 patients (55.4%) were aged above 60 years. Two age peaks were observed at 0–9 years and 60–69 years (Fig. 4). The annual number of patients differed slightly; however, on average, 645 patients were treated by HEMS every year. When restricted to HEMS patients within Tochigi prefecture (excluding patients in two neighboring prefectures by virtue of the agreement of wide-area HEMS cooperation), the crude rate was 31.7 patients per 100,000 people (Fig. 3), which corresponds to the age-standardized rate of 30.3 patients per 100,000 people.

**Table 2:** Demographics and clinical characteristics of patients (N = 5163) treated by the Tochigi Helicopter Emergency Medical Services

	n (%) or mean ± SD			
	Air lifted	Ground escorted	Assisted	Total
	n = 4215	n = 548	n = 400	n = 5163
*Sex*				
Men	2874 (68.2)	356 (65.0)	259 (64.8)	3489 (67.6)
*Age (in years; mean* ± *SD)*	55.2 ± 24.5	57.7 ± 24.5	58.6 ± 26.1	55.7 ± 24.7
0–9	342 (8.1)	36 (6.6)	19 (4.8)	397 (7.7)
10–19	223 (5.3)	25 (4.6)	42 (10.5)	290 (5.6)
20–29	193 (4.6)	27 (4.9)	12 (3.0)	232 (4.5)
30–39	226 (5.4)	28 (5.1)	22 (5.5)	276 (5.3)
40–49	391 (9.3)	60 (10.9)	31 (7.8)	482 (9.3)
50–59	540 (12.8)	53 (9.7)	37 (9.3)	630 (12.2)
60–69	892 (21.2)	105 (19.2)	60 (15.0)	1057 (20.5)
70–79	825 (19.6)	104 (19)	87 (21.8)	1016 (19.7)
80–89	525 (12.5)	93 (17)	62 (15.5)	680 (13.2)
90–99	58 (1.4)	17 (3.1)	27 (6.8)	102 (2.0)
100 >	0 (0.0)	0 (0.0)	1 (0.3)	1 (0.0)
*Year*				
2012	483 (11.5)	68 (12.4)	33 (8.3)	584 (11.3)
2013	491 (11.6)	69 (12.6)	20 (5.0)	580 (11.2)
2014	552 (13.1)	78 (14.2)	42 (10.5)	672 (13.0)
2015	612 (14.5)	88 (16.1)	54 (13.5)	754 (14.6)
2016	553 (13.1)	59 (10.8)	67 (16.8)	679 (13.2)
2017	501 (11.9)	62 (11.3)	42 (10.5)	605 (11.7)
2018	514 (12.2)	56 (10.2)	71 (17.8)	641 (12.4)
2019	509 (12.1)	68 (12.4)	71 (17.8)	648 (12.6)
*Season*				
Spring (March–May)	1112 (26.4)	122 (22.3)	89 (22.3)	1323 (25.6)
Summer (June–August)	1112 (26.4)	130 (23.7)	119 (29.8)	1361 (26.4)
Autumn (September–November)	1029 (24.4)	130 (23.7)	100 (25.0)	1259 (24.4)
Winter (December-February)	962 (22.8)	166 (30.3)	92 (23.0)	1220 (23.6)
*Median Distance (in km) (IQR)*	27.4 (15.9–37.7)	25.4 (14.3–34.4)	29.7 (18.9–38.3)	27.4 (15.8–37.1)
Missing	110 (2.6)	16 (2.9)	26 (6.5)	152 (2.9)
*Time from helicopter takeoff to flight doctor encounter, min mean* ± *SD*	14.5 ± 12.7	15.8 ± 17.8	15.1 ± 6.6	14.7 ± 13.0
Missing	119 (2.8)	43 (7.8)	48 (12.0)	210 (4.1)
*Diagnosis*				
Trauma	1490 (35.3)	145 (26.5)	111 (27.8)	1746 (33.8)
Neurology	395 (9.4)	70 (12.8)	47 (11.8)	512 (9.9)
Cardiovascular diseases	1385 (32.9)	167 (30.5)	74 (18.5)	1,626 (31.5)
Ischemic heart disease	379 (9.0)	40 (7.3)	6 (1.5)	425 (8.2)
Aortic disease	94 (2.2)	4 (0.7)	3 (0.8)	101 (2.0)
Stroke	772 (18.3)	103 (18.8)	54 (13.5)	929 (18.0)
Others	140 (3.3)	20 (3.6)	11 (2.8)	171 (3.3)
Cardiopulmonary arrest	99 (2.3)	34 (6.2)	10 (2.5)	143 (2.8)
Respiratory disease	70 (1.7)	7 (1.3)	12 (3.0)	89 (1.7)
Gastroenterology	113 (2.7)	11 (2.0)	17 (4.3)	141 (2.7)
Allergies	163 (3.9)	18 (3.3)	15 (3.8)	196 (3.8)
Toxicosis	58 (1.4)	16 (2.9)	3 (0.8)	77 (1.5)
Other diseases	442 (10.5)	80 (14.6)	111 (27.8)	633 (12.3)
*Pre-hospital severity*				
Mild	494 (11.7)	83 (15.1)	147 (36.8)	724 (14.0)
Moderate	1221 (29.0)	193 (35.2)	156 (39.0)	1570 (30.4)
Severe	2485 (59.0)	265 (48.4)	86 (21.5)	2,836 (54.9)
Death	7 (0.2)	7 (1.3)	1 (0.3)	15 (0.3)
Missing	8 (0.2)	0 (0)	10 (2.5)	18 (0.3)
*Pre-hospital GCS*				
3–8	896 (21.3)	152 (27.7)	51 (12.8)	1099 (21.3)
9–13	749 (17.8)	93 (17.0)	64 (16.0)	906 (17.5)
14–15	2535 (60.1)	297 (54.2)	271 (67.8)	3103 (60.1)
Missing	35 (0.8)	6 (1.1)	14 (3.5)	55 (1.1)
*Pre-hospital interventions*				
Intravenous drip	3954 (93.8)	509 (92.9)	345 (86.3)	4808 (93.1)
Oxygen administration	3185 (75.6)	378 (69.0)	191 (47.8)	3754 (72.7)
Ultrasound examination	2513 (59.6)	296 (54.0)	226 (56.5)	3035 (58.8)
Endotracheal intubation	584 (13.9)	60 (10.9)	7 (1.8)	651 (12.6)
Pleural drainage	71 (1.7)	6 (1.1)	1 (0.3)	78 (1.5)
Chest compression	96 (2.3)	32 (5.8)	5 (1.3)	133 (2.6)
External defibrillation	145 (3.4)	19 (3.5)	4 (1.0)	168 (3.3)

With regard to diagnosis, trauma and cardiovascular disease were the two most common diseases, accounting for 65.3% of the total number of cases (Table 2). Stroke and ischemic heart disease accounted for 83.2% of the total number of cardiovascular diseases. As shown in the disease-specific age distributions (Fig. 4), the first age peak observed at 0–9 years was mainly attributable to neurological diseases, and seizures accounted for 80.5% of the patients in this group. The second age peak observed at 60–70 years was mainly attributable to cardiovascular disease.

### Geographical and environmental variables

The median distance from the base hospital to the site was 26.7 km. The number of patients was similar across the four seasons (Table 2). Patients in the base hospital medical control areas (D, E, and I) accounted for 75.3% of the total number of patients (Fig. 3), resulting in the highest annual case-rates in the same areas. Interestingly, patients in area C, which was not a base hospital control area, were treated by the HEMS as frequently as those in the base hospital medical control areas. Additionally, the percentage of patients that were air lifted from area C was higher than that in the base hospital medical control areas (Fig. 3).

### Pre-hospital intervention, mode and type of transport, and short-term outcome

**Fig. 5:**
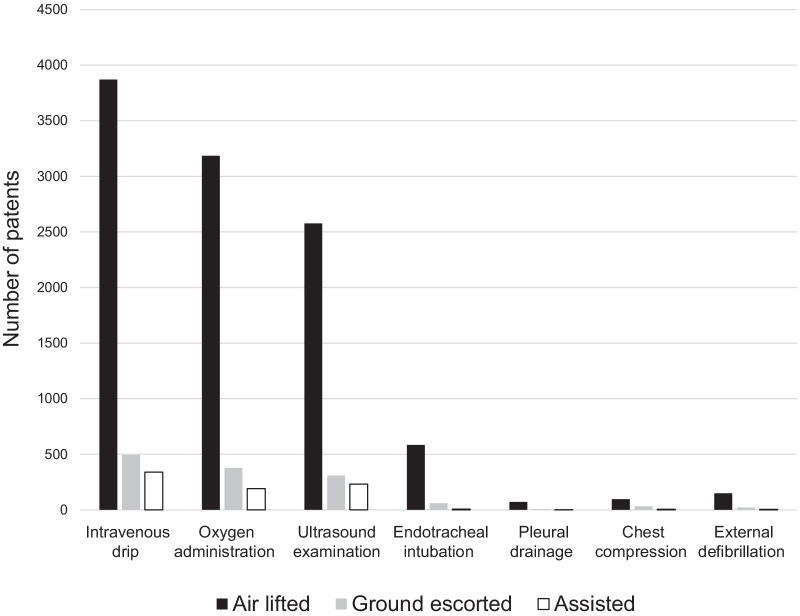
Mission-wise distribution of pre-hospital interventions. The numbers of each pre-hospital intervention among air lifted, ground escorted, and assisted patients were, respectively, as follows: intravenous drip (3870, 494, and 339), oxygen administration (3185, 377, and 191); ultrasound examination (2576, 308, and 231); endo-tracheal intubation (584, 60, and 7); pleural drainage (71, 6, and 1); chest compression (96, 32, and 5); external defibrillation (149, 21, and 4)

Intravenous drip (93.1%) and oxygen administration (72.7%) were the most commonly performed pre-hospital interventions (Table 2). Ultrasound examination and endotracheal intubation, which are pre-hospital interventions, were performed for 58.8% and 12.6% of patients, respectively (Fig. 5).

Of the HEMS patients treated on-scene, 81.6% were transported by the helicopter ambulance after immediate pre-hospital intervention (53.4% and 28.2% to the base hospital and other hospitals, respectively; Fig. 1 and Table 2). Air lifted patients were transported mainly to the base hospital (65.4% of all the air lifted patients), whereas ground escorted patients were transported primarily to other hospitals (69.5% of all the ground escorted patients; see Additional file 1: Table S1). The distance for cases transported to the base hospital was shorter than that for the cases transported to other hospitals for both air lifted and ground escorted transportation (see Additional file 1: Table S1).

Additionally, 56.6% of overall HEMS patients were transported to the base hospital (Fig. 1); the overall recovery rate was 77.4% for patients transported to the base hospital in the supplementary data analysis (see Additional file 1: Table S2).

We also briefly summarized published studies to compare HEMS systems in Japan and other countries (see Additional file 1: Table S3).

## Discussion

We aimed to examine the overall characteristics of patients who were received HEMS in Tochigi, Japan. We found that the HEMS was requested approximately 2.5 times per day on an average. The age-standardized rate of treatment of the HEMS patients within this prefecture was 30.3 patients per 100,000 people; it did not differ significantly from the crude rate (31.7). This suggests that our data from the survey of Tochigi HEMS, where the population is representative of the Japanese population, may posit a certain level of generalizability to the national population. The most common disease was trauma (33.8%), followed by cardiovascular diseases (31.5%) with two age peaks observed at 0–9 and 60–69 years. The number of HEMS patients differed across official emergency rescue activity areas. More than 90% of the patients were administered intravenous infusion, and approximately 60% of the patients underwent immediate ultrasound examination. Most HEMS patients (81.6%) were transported by a helicopter ambulance after immediate pre-hospital intervention. In addition, regardless of the mode and type of transport, 56.6% of HEMS patients were transported to the base hospital, and their short-term recovery rates were greater than 75% in the supplementary data analysis.

Similar to other countries [13, 14, 17–19], we found that trauma and cardiovascular diseases are most common cases encountered by the HEMS, particularly among male patients in Japan; this suggests that HEMS needs might differ across local and/or national settings. For these patients, reduced time for hospital transportation on account of the helicopter ambulance is the key to better outcomes, particularly when distant transportation is required [20–24]. In a comparative study conducted in the U.S.A., the mortality rate was lower among HEMS patients than for the ground emergency medical services patients [20]. Additionally, although the ambulance staff of ground emergency medical services in Japan are generally not allowed to provide advanced medical care, all the HEMS patients can be provided with immediate pre-hospital intervention by onboard physicians. As briefly summarized in Additional file 1: Table S3, although Japanese HEMS has a short history compared to other international settings and does not dispatch at night, a higher density of available helicopter ambulances in this small, overpopulated country is likely to be highlighted. Therefore, in the current study, the high percentage of patients with trauma and cardiovascular diseases, which were requested by the local communications command section of the fire department, is reasonable.

The two age peaks observed in the current study are in line with findings from other countries [13, 14, 19]. This pattern would be reasonable for common age-specific emergencies, such as neurological and cardiovascular diseases. For the peak in the younger age group (0–9 years), most of the emergency requests were due to seizures; this pattern has also been observed in previous studies in Japan [11, 25]. In addition, the typical onset of cardiovascular diseases among older adults might explain the second peak (60–69 years); previous studies have reported that the peak age of patients with cardiovascular diseases who were transported by the HEMS was approximately 65 years [19].

The rate of HEMS patients differed across regional medical control areas. This finding may imply plausible differences in access to tertiary emergency centers. For instance, although the (straight-line) distance is relatively shorter from the base hospital to the base hospital control areas (D, E, and I) as compared to the other areas, these are mountainous districts; therefore, the HEMS might be frequently requested because transporting patients via automotive ambulance takes time due to terrain and road problems. Frequent communication between the emergency physicians at the base hospital and emergency paramedical staff in the base hospital control areas might also play a potential role in easing HEMS requests. In addition, access to emergency medical centers from area C is relatively inconvenient as compared to that from other neighboring areas in the prefecture. In these ground emergency medical services less-advantaged areas, the high annual rate of emergency cases and high percentages of patients air lifted by the HEMS is reasonable. We believe that our findings may partly reflect the effective use of helicopter ambulances to mitigate geographical disparities in emergency medical resources.

Regarding pre-hospital interventions, it was observed that more than 90% of the patients were administered intravenous infusion, and ultrasound examination was performed for approximately 60% of the HEMS patients. In addition to maintaining hemodynamics and administering pharmaceutical agents via intravenous infusions during emergencies, ultrasound examinations play a critical role. The immediate identification of blood loss, pneumothorax, and cardiovascular diseases via ultrasound examinations is known to be effective for patients with trauma and cardiovascular diseases in prehospital settings [26]. Unlike previous studies, endotracheal intubation was observed to be less common in the current study. While the percentage of critically severe patients (defined as NACA score 4–7) was 61% in Denmark [13], the percentage of critically severe patients (according to our study criteria) in the current study was approximately 55%. Although endotracheal intubation is known to benefit critically ill patients [27, 28], provision of the best respiratory treatment—depending on the physician’s proficiency, patient condition, and topography—should be a priority [29]. Because the Tochigi HEMS covers a relatively small area as compared to the HEMS in other settings [11], it is plausible that the emergency physicians might have prioritized immediate transport without the implementation of endotracheal intubation in certain cases.

This study had several limitations. First, this study evaluated only one local prefecture, thereby limiting the generalizability of data. However, because the study utilized a complete survey of HEMS patients in the prefecture, we were able to use standardized statistical tools to analyze characteristics of the HEMS patients; this can be useful in comparing cases across settings. Second, data for some parameters were missing; we did not perform analytical epidemiology, which is beyond the scope of the current study. Additionally, data for the ground emergency medical services patients were not available. Thus, conclusions regarding the pros and cons of the Japanese HEMS system could not be drawn. Third, data for patients who were transported to other prefectures were not available; thus, the number of HEMS patients in Tochigi Prefecture might have been underestimated. Fourth, although the severity was subjectively determined by the onboard physicians, detailed and accurate severity and/or emergency criteria (such as the NACA score and the Canadian Triage and Acuity Scale, or the Japan Triage and Acuity Scale) were not available [30]. In addition, relevant short- and long-term clinical outcomes (e.g., 30-day in-hospital mortality and 90-day mortality) were not assessed. Therefore, future studies should objectively assess the relevant severity of emergencies and clinical outcomes in Japan.

Despite these limitations, our strengths included the study size; this is one of the largest Japanese HEMS studies. Furthermore, to the best of our knowledge, our study is the first to document the overall characteristics of HEMS patients in Japan. To expedite further understanding of the current HEMS situation in Japan, future research using national-level data (such as that published in the Japanese Society for Aeromedical Services Registry; https://square.umin.ac.jp/jsas/) and further analyses using local-level data (which might yield perspectives that are different from the national-level registry data) are warranted.

## Conclusions

This study is the first to describe the overall characteristics of the HEMS patients using comprehensive data of all the HEMS patients over eight years (2012–2019) in Tochigi, Japan. We analyzed the demographic, geographical, and clinical characteristics of the HEMS patients; further research using both local- and national-level data is needed to accelerate the understanding of the HEMS’ benefits.

## Supplementary Information

**Additional file 1. Table S1**: Demographics and clinical characteristics of patients across different modes and types of transport. This data represents the 5163 patient cases treated by the Tochigi Helicopter Emergency Medical Services. In addition to Table 2 in the main text, the different characteristics of patients who were transported to the base hospital and to other hospitals are further described in this data. **Table S2**: Demographics and characteristics of patients (N = 2924) transported to the base hospital by the Tochigi helicopter emergency medical service. This is the data of 2924 cases of patients transported to the base hospital. In addition to Table 2 in the main text, short-term clinical outcomes are described in this data. **Table S3**: International variations of backgrounds and outcomes of helicopter emergency medical services. This data briefly summarizes international reports of the backgrounds and outcomes of helicopter emergency medical services.

## Data Availability

The datasets used and/or analyzed during the current study are available from Dr. Koji Wake on reasonable request. Restrictions apply to the availability of these data, which were used under license for this study, and so are not publicly available.

## Abbreviations

HEMS: Helicopter Emergency Medical Services
MHLW: Ministry of Health, Labour and Welfare
ICD-10: International Classification of Diseases, 10th revision
GCS: Glasgow Coma Scale
NACA: National Advisory Committee on Aeronautics
