# Supplementary material for: Characteristics of patients who received helicopter emergency medical services in Japan from 2012 to 2019: a retrospective analysis of data from Tochigi Prefecture
Source: Scand J Trauma Resusc Emerg Med. 2022 Apr 11;30:25. doi: 10.1186/s13049-022-01012-6 (PMC8996593; doi:10.1186/s13049-022-01012-6)
Supplement: Supplementary file 1 — Additional file 1. Table S1: Demographics and clinical characteristics of patients across different modes and types of transport. This data represents the 5163 patient cases treated by the Tochigi Helicopter Emergency Medical Services. In addition to Table 2 in the main text, the different characteristics of patients who were transported to the base hospital and to other hospitals are further described in this data. Table S2: Demographics and characteristics of patients (N = 2924) transported to the base hospital by the Tochigi helicopter emergency medical service. This is the data of 2924 cases of patients transported to the base hospital. In addition to Table 2 in the main text, short-term clinical outcomes are described in this data. Table S3: International variations of backgrounds and outcomes of helicopter emergency medical services. This data briefly summarizes international reports of the backgrounds and outcomes of helicopter emergency medical services. [file 13049_2022_1012_MOESM1_ESM.pdf]

Table S1. Characteristics of patients stratified by transportation types and modes

Characteristics	N (%) or mean ± SD					
	Air lifted		Ground escorted		Assisted	Total
	Base hospital n = 2757	Other hospitals n =1458	Base hospital n = 167	Other hospitals n = 381	n = 400	n = 5163
**Men**	1851 (67.1)	1023 (70.2)	104 (62.3)	252 (66.1)	259 (64.8)	3489 (67.6)
**Age**, years	54.0±25.3	57.2±22.8	49.8±26.9	61.2±22.6	58.6±26.1	55.69±24.7
0-9	267 (9.7)	75 (5.1)	22 (13.2)	14 (3.7)	19 (4.8)	397 (7.7)
10-19	165 (6.0)	58 (4.0)	12 (7.2)	13 (3.4)	42 (10.5)	290 (5.6)
20-29	112 (4.1)	81 (5.6)	11 (6.6)	16 (4.2)	12 (3.0)	232 (4.5)
30-39	138 (5.0)	88 (6.0)	7 (4.2)	21 (5.5)	22 (5.5)	276 (5.3)
40-49	252 (9.1)	139 (9.5)	16 (9.6)	44 (11.5)	31 (7.8)	482 (9.3)
50-59	355 (12.9)	185 (12.7)	18 (10.8)	35 (9.2)	37 (9.3)	630 (12.2)
60-69	572 (20.7)	320 (21.9)	33 (19.8)	72 (18.9)	60 (15.0)	1057 (20.5)
70-79	525 (19.0)	300 (20.6)	27 (16.2)	77 (20.2)	87 (21.8)	1016 (19.7)
80-89	343 (12.4)	182 (12.5)	21 (12.6)	72 (18.9)	62 (15.5)	680 (13.2)
90-99	28 (1.0)	30 (2.1)	0 (0.0)	17 (4.5)	27 (6.8)	102 (2.0)
>100	0 (0.0)	0 (0.0)	0 (0.0)	0 (0.0)	1 (0.3)	1 (0.0)
**Year**						
2012	323 (11.7)	160 (11.0)	12 (7.2)	56 (14.7)	33 (8.3)	584 (11.3)
2013	316 (11.5)	175 (12.0)	10 (6.0)	59 (15.5)	20 (5.0)	580 (11.2)
2014	387 (14.0)	165 (11.3)	18 (10.8)	60 (15.7)	42 (10.5)	672 (13.0)
2015	423 (15.3)	189 (13.0)	32 (19.2)	56 (14.7)	54 (13.5)	754 (14.6)
2016	372 (13.5)	181 (12.4)	31 (18.6)	28 (7.3)	67 (16.8)	679 (13.2)
2017	323 (11.7)	178 (12.2)	22 (13.2)	40 (10.5)	42 (10.5)	605 (11.7)
2018	315 (11.4)	199 (13.6)	20 (12.0)	36 (9.4)	71 (17.8)	641 (12.4)
2019	298 (10.8)	211 (14.5)	22 (13.2)	46 (12.1)	71 (17.8)	648 (12.6)
**Season**						
Spring (March-May)	732 (26.6)	380 (26.1)	34 (20.4)	88 (23.1)	89 (22.3)	1323 (25.6)
Summer (June-August)	746 (27.1)	366 (25.1)	41 (24.6)	89 (23.4)	119 (29.8)	1361 (26.4)
Autumn (September-November)	658 (23.9)	371 (25.4)	35 (21.0)	95 (24.9)	100 (25.0)	1259 (24.4)
Winter (December-February)	621 (22.5)	341 (23.4)	57 (34.1)	109 (28.6)	92 (23.0)	1220 (23.6)
**Median distance (IQR)**, km	20.9 (13.9-31.1)	34.4 (27.8-45.3)	15.8 (12.7-25.5)	29.0 (15.3-36.9)	29.1 (16.0-37.2)	26.7 (15.1-36.6)
Missing	57 (2.1)	53 (3.6)	4 (2.4)	12 (3.1)	26 (6.5)	152 (2.9)
**Time from helicopter takeoff to flight doctor encounter**, min	13.2±6.7	17.1±19.1	14.4±9.4	16.4±20.1	15.1±6.6	14.7±13.0
Missing	86 (3.1)	33 (2.3)	23 (13.8)	20 (5.2)	48 (12.0)	210 (4.1)
**Diagnosis**						
Trauma	839 (30.4)	651 (44.7)	49 (29.3)	96 (25.2)	111 (27.8)	1746 (33.8)
Neurology	309 (11.2)	86 (5.9)	28 (16.8)	42 (11.0)	47 (11.8)	512 (9.9)
Cardiovascular diseases	935 (33.9)	450 (30.9)	44 (26.3)	123 (32.3)	74 (18.5)	1,626 (31.5)
Ischemic heart disease	266 (9.6)	113 (7.8)	15 (9.0)	25 (6.6)	6 (1.5)	425 (8.2)
Aortic disease	65 (2.4)	29 (2.0)	2 (1.2)	2 (0.5)	3 (0.8)	101 (2.0)
Stroke	502 (18.2)	270 (18.5)	23 (13.8)	80 (21)	54 (13.5)	929 (18.0)
Others	102 (3.7)	38 (2.6)	4 (2.4)	16 (4.2)	11 (2.8)	171 (3.3)
Cardiopulmonary arrest	77 (2.8)	22 (1.5)	6 (3.6)	28 (7.3)	10 (2.5)	143 (2.8)
Respiratory disease	50 (1.8)	20 (1.4)	4 (2.4)	3 (0.8)	12 (3.0)	89 (1.7)
Gastroenterology	89 (3.2)	24 (1.6)	2 (1.2)	9 (2.4)	17 (4.3)	141 (2.7)
Allergies	100 (3.6)	63 (4.3)	8 (4.8)	10 (2.6)	15 (3.8)	196 (3.8)
Toxicosis	45 (1.6)	13 (0.9)	10 (6.0)	6 (1.6)	3 (0.8)	77 (1.5)
Other diseases	313 (11.4)	129 (8.8)	16 (9.6)	64 (16.8)	111 (27.8)	633 (12.3)
**Pre-hospital severity**						
Mild	399 (14.5)	95 (6.5)	30 (18.0)	53 (13.9)	147 (36.8)	724 (14.0)
Moderate	741 (26.9)	480 (32.9)	55 (32.9)	138 (36.2)	156 (39.0)	1570 (30.4)
Severe	1,608 (58.3)	877 (60.2)	82 (49.1)	183 (48)	86 (21.5)	2,836 (54.9)
Death	5 (0.2)	2 (0.1)	0 (0.0)	7 (1.8)	1 (0.3)	15 (0.3)
Missing	4 (0.1)	4 (0.3)	0 (0.0)	0 (0.0)	10 (2.5)	18 (0.3)
**Pre-hospital GCS**						
3-8	651 (23.6)	245 (16.8)	43 (25.7)	109 (28.6)	51 (12.8)	1099 (21.3)
9-13	497 (18.0)	252 (17.3)	35 (21.0)	58 (15.2)	64 (16.0)	906 (17.5)
14-15	1580 (57.3)	955 (65.5)	85 (50.9)	212 (55.6)	271 (67.8)	3103 (60.1)
Missing	29 (1.1)	6 (0.4)	4 (2.4)	2 (0.5)	14 (3.5)	55 (1.1)
**Pre-hospital interventions**						
Intravenous drip	2547 (92.4)	1407 (96.5)	148 (88.6)	361 (94.8)	345 (86.3)	4808 (93.1)
Oxygen administration	2070 (75.1)	1,115 (76.5)	114 (68.3)	264 (69.3)	191 (47.8)	3754 (72.7)
Ultrasound examination	1553 (56.3)	960 (65.8)	81 (48.5)	215 (56.4)	226 (56.5)	3035 (58.8)
Endotracheal intubation	415 (15.1)	169 (11.6)	20 (12.0)	40 (10.5)	7 (1.8)	651 (12.6)
Pleural drainage	43 (1.6)	28 (1.9)	2 (1.2)	4 (1.0)	1 (0.3)	78 (1.5)
Chest compression	79 (2.9)	17 (1.2)	10 (6.0)	22 (5.8)	5 (1.3)	133 (2.6)
External defibrillation	113 (4.1)	32 (2.2)	7 (4.2)	12 (3.1)	4 (1.0)	168 (3.3)

Table S2. Characteristics of patients (N = 2924) transported to the base hospital

Characteristics	N (%) or mean ± SD		
	**Air lifted** (n = 2757)	**Ground escorted** (n = 167)	**Total** (n = 2924)
**Men**	1851 (67.1)	104 (62.3)	1955 (66.9)
**Age**, years	54.0±25.3	49.8±26.9	53.8±25.4
0-9	267 (9.7)	22 (13.2)	289 (9.9)
10-19	165 (6.0)	12 (7.2)	177 (6.1)
20-29	112 (4.1)	11 (6.6)	123 (4.2)
30-39	138 (5.0)	7 (4.2)	145 (5.0)
40-49	252 (9.1)	16 (9.6)	268 (9.2)
50-59	355 (12.9)	18 (10.8)	373 (12.8)
60-69	572 (20.7)	33 (19.8)	605 (20.7)
70-79	525 (19.0)	27 (16.2)	552 (18.9)
80-89	343 (12.4)	21 (12.6)	364 (12.4)
90-99	28 (1.0)	0 (0.0)	28 (1.0)
**Year**			
2012	323 (11.7)	12 (7.2)	335 (11.5)
2013	316 (11.5)	10 (6.0)	326 (11.1)
2014	387 (14.0)	18 (10.8)	405 (13.9)
2015	423 (15.3)	32 (19.2)	455 (15.6)
2016	372 (13.5)	31 (18.6)	403 (13.8)
2017	323 (11.7)	22 (13.2)	345 (11.8)
2018	315 (11.4)	20 (12.0)	335 (11.5)
2019	298 (10.8)	22 (13.2)	320 (10.9)
**Season**			
Spring (March-May)	732 (26.6)	34 (20.4)	766 (26.2)
Summer (June-August)	746 (27.1)	41 (24.6)	787 (26.9)
Autumn (September-November)	658 (23.9)	35 (21.0)	693 (23.7)
Winter (December-February)	621 (22.5)	57 (34.1)	678 (23.2)
Median distance (IQR), km	20.9 (13.9-31.1)	15.8 (12.7-25.5)	20.6 (13.8-31.0)
Missing	57 (2.1)	4 (2.4)	61 (2.1)
**Time from helicopter takeoff to flight doctor encounter**, min	13.2±6.7	14.4±9.4	13.4±7.4
Missing	86 (3.1)	23 (13.8)	109 (3.7)
**Diagnosis**			
Trauma	839 (30.4)	49 (29.3)	888 (30.4)
Neurology	309 (11.2)	28 (16.8)	337 (11.5)
Cardiovascular diseases	935 (33.9)	44 (26.3)	979 (33.5)
Ischemic heart disease	266 (9.6)	15 (9.0)	281 (9.6)
Aortic disease	65 (2.4)	2 (1.2)	67 (2.3)
Stroke	502 (18.2)	23 (13.8)	525 (18.0)
Others	102 (3.7)	4 (2.4)	106 (3.6)
Cardiopulmonary arrest	77 (2.8)	6 (3.6)	83 (2.8)
Respiratory disease	50 (1.8)	4 (2.4)	54 (1.8)
Gastroenterology	89 (3.2)	2 (1.2)	91 (3.1)
Allergies	100 (3.6)	8 (4.8)	108 (3.7)
Toxicosis	45 (1.6)	10 (6.0)	55 (1.9)
Other diseases	313 (11.4)	16 (9.6)	329 (11.3)
**Pre-hospital severity**			
Mild	399 (14.5)	30 (18.0)	429 (14.7)
Moderate	741 (26.9)	55 (32.9)	796 (27.2)
Severe	1608 (58.3)	82 (49.1)	1690 (57.8)
Death	5 (0.2)	0 (0.0)	5 (0.2)
Missing	4 (0.1)	0 (0.0)	4 (0.1)
**Pre-hospital GCS**			
3-8	651 (23.6)	43 (25.7)	694 (23.7)
9-13	497 (18.0)	35 (21.0)	532 (18.2)
14-15	1,580 (57.3)	85 (50.9)	1,665 (56.9)
Missing	29 (1.1)	4 (2.4)	33 (1.1)
**Pre-hospital interventions**			
Intravenous drip	2547 (92.4)	148 (88.6)	2695 (92.2)
Oxygen administration	2070 (75.1)	114 (68.3)	2184 (74.7)
Ultrasound examination	1553 (56.3)	81 (48.5)	1634 (55.9)
Endotracheal intubation	415 (15.1)	20 (12.0)	435 (14.9)
Pleural drainage	43 (1.6)	2 (1.2)	45 (1.5)
Chest compression	79 (2.9)	10 (6.0)	89 (3.0)
External defibrillation	113 (4.1)	7 (4.2)	120 (4.1)
**Short-term clinical outcomes**			
Recovering	2125 (77.1)	137 (82)	2262 (77.4)
No changed	138 (5.0)	5 (3.0)	143 (4.9)
Worse	11 (0.4)	0 (0.0)	11 (0.4)
Death	289 (10.5)	15 (9)	304 (10.4)
Missing	194 (7.0)	10 (6.0)	204 (7.0)

Table S3. International variations of backgrounds and outcomes of helicopter emergency medical services

Country	HEMS system backgrounds and settings†							Reported characteristics and outcomes			
	Year started	No. of helicopter ambulance				Flight conditions	GDP (USD, billion)	Study patients	Endotracheal Intubation rate	Main diseases	Clinical outcomes
		Total	Per 100,000 population	Per 10000 km^2	Doctor onboard						
Germany[1]	1970	89	83.1	2.49	88	Daytime / Night (partly)	3,861	Trauma[4] (ISS >9)	HEMS 65.7% GEMS 40,6%	N.A.	The mortality OR in HEMS compared to GEMS was 0.75.
Switzerland[1]	1952	40	8.6	9.69	23	Daytime / Night (partly)	732	Patients rescued by helicopter hoist operation[5]	Day 2% Night 1%	Trauma (64.5%)	Nearly 20 % of patients who needed to be evacuated by a hoist were severely injured, and complex and lifesaving medical interventions were necessary before the helicopter hoist operation.
France[1]	1983	63	67.3	1.16	12	Daytime / Night (partly)	2,716	Trauma[6]	HEMS 14% GEMS 14%	N.A.	Using the TRISS predictive score of mortality, the standardized mortality ratio was lower in the HEMS group than in the GEMS group.
Norway[1]	1977	13	5.3	0.34	13	Daytime / All night	406	All HEMS[7]	N.A.	N.A.	Treatment prior to HEMS arrival reduced on-scene time in patients with acute myocardial infarction/stroke.
Finland[1]	2011	6	5.5	0.18	6	Daytime / All night	269	All HEMS[8]	N.A.	Trauma (26%) Cardiac arrest (20%)	N.A.
Denmark[1]	2010	3	5.8	0.70	3	Daytime / All night	350	All HEMS[9]	HEMS 21%	Cardiovascular (41%) Trauma (23%) Neurology (16%)	N.A.
Sweden[1]	1970	10	10.3	0.22	8	Daytime / Night (partly)	531	All HEMS[10]	HEMS 17%	Trauma (40%) Chest pain (11.4%) Cardiac arrest (10.9%)	N.A.
Italy[1]	1952	33	59.7	1.10	2	Daytime / Night (partly)	2,005	Traumatic cardiac arrest[11] (ISS ≥16)	N.A.	N.A.	3.6% survival with HEMS vs. 0% survival with GEMS (not significant)
UK[1]	1989	35	66.8	1.44	24	Daytime / Night (partly)	2,831	All HEMS[12] (one hospital)	HEMS 35%	Trauma (51%)	N.A.
USA[2][16]	1972	386	328.3	0.39	Unknown	Daytime / All night	21,430	Trauma[13]	N.A.	N.A.	HEMS patients were 57.0% less likely to die than GEMS patients.
Japan[2]	2001	54	126.3	1.43	54	Daytime only	5,065	Trauma[14]	HEMS 5.8% GEMS 40.6%	N.A.	The survival rates of HEMS patients were significantly greater than those transported by GEMS (OR 1.23).
								All HEMS (The present study)	HEMS 12.6%	Trauma (33.8%) Cardiovascular (31.5%) Neurology (9.9%)	The short-term recovery rate was >75% among those transported to the base hospital.
South Africa[3]	1976	8	58.6	0.07	8	Daytime / All night	351	All HEMS[15]	N.A.	Interfacility transfers: Obstetric (32.1%) Pediatric (25.7%) Trauma (21.8%)	Most flights (88.4%) were interfacility transfers.

Abbreviation: HEMS, helicopter emergency medical services; GEMS, ground emergency medical services; ISS, Injury Severity Score; TRISS, Trauma and Injury Severity Score. N.A.; not available/not applicable.
† Data for population and GDP were for 2019. The backgrounds and settings were extracted from previous studies. Data for official HEMS flight ranges for each country were not available.

The reference list for Table S3 is below:

1. Jones A, Donald MJ, Jansen JO. Evaluation of the provision of helicopter emergency medical services in Eur ope. Emerg Med J. 2018;35:720-5.
2. Emergency Medical Network of Helicopter and Hospital (Nonprofit organization): Doctor Helicopter service began in Japan after overseas countries. https://hemnet.jp/en-know-history (2020). Accessed 20 Nov 2021.
3. S.A. RED CROSS AIR MERCY SERVICE: Changing lives. AMS ANNUAL REPORT 2021. https://ams.o rg.za/annual-report/. Accessed 25 Feb 2022.
4. Andruszkow H, Lefering R, Frink M, Mommsen P, Zeckey C, Rahe K, et al. Survival benefit of helicopter em ergency medical services compared to ground emergency medical services in traumatized patients. Crit Care. 2013;17:R124.
5. Pietsch U, Knapp J, Mann M, Meuli L, Lischke V, Tissi M, et al. Incidence and challenges of helicopter emer gency medical service (HEMS) rescue missions with helicopter hoist operations: analysis of 11,228 daytime a nd nighttime missions in Switzerland. Scand J Trauma Resusc Emerg Med. 2021;29:92.
6. Ageron F, Debaty G, Savary D, Champly F, Albasini F, Usseglio P, et al. Association of helicopter transporta tion and improved mortality for patients with major trauma in the northern French Alps trauma system: an ob servational study based on the TRENAU registry. Scand J Trauma Resusc Emerg Med. 2020;28:35.
7. Østerås Ø, Heltne JK, Vikenes BC, Assmus J, Brattebø G. Factors influencing on-scene time in a rural Norwe gian helicopter emergency medical service: a retrospective observational study. Scand J Trauma Resusc Emer g Med. 2017;25:97.
8. Saviluoto A, Björkman J, Olkinuora A, Virkkunen I, Kirves H, Setälä P, et al. The first seven years of nationa lly organized helicopter emergency medical services in Finland- the data from quality registry. Scand J Traum a Resusc Emerg Med. 2020;28:46.
9. Alstrup K, Møller TP, Knudsen L, Hansen TM, Petersen JAK, Rognås L, et al. Characteristics of patients trea ted by the Danish Helicopter Emergency Medical Service from 2014-2018: a nationwide population-based stu dy. Scand J Trauma Resusc Emerg Med. 2019;27:102.
10. Kornhall D, Näslund R, Klingberg C, Schiborr R, Gellerfors M. The mission characteristics of a newly impl emented rural helicopter emergency medical service. BMC Emerg Med. 2018;18:28.
11. Di Bartolomeo S, Sanson G, Nardi G, Michelutto V, Scian F. HEMS VS. GROUND-BLS CARE IN TRAU MATIC CARDIAC ARREST. Prehosp Emerg Care. 2005;9:79-84.
12. Catherall JA, Brown A, Benger JR. Characteristics of patients transported by an air ambulance critical care t eam. Emerg Med J. 2013;30:419-20.
13. Michaels D, Pham H, Puckett Y, Dissanaike S. Helicopter versus ground ambulance: review of national data base for outcomes in survival in transferred trauma patients in the USA. Trauma Surg Acute Care Open. 201 9;4:e000211.
14. Abe T, Takahashi O, Saitoh D, Tokuda Y. Association between helicopter with physician versus ground em ergency medical services and survival of adults with major trauma in Japan. Crit Care. 2014;18:R146.
15. Wood D, D'Andrea PA, Smith WP, Van Hoving DJ. A 5-year analysis of the helicopter air mercy service in Richards Bay, South Africa. South African Medical Journal. 2014;104:124-6.
16. Emergency Transport Healthcare Operations and Services: Air Medical Base Dashboard. https://ethos.aams. org/. Accessed 25 Feb 2022.
